# Supplementary material for: Secondary Genetic Events and Their Relationship to TP53 Mutation in Mantle Cell Lymphoma: A Sub-Study from the FIL_MANTLE-FIRST BIO on Behalf of Fondazione Italiana Linfomi (FIL)
Source: Cancers (Basel). 2025 Dec 17;17(24):4027. doi: 10.3390/cancers17244027 (PMC12731303; doi:10.3390/cancers17244027)
Supplement: Supplementary file 1 [file cancers-17-04027-s001.zip › cancers-4028565-supplementary.pdf]

Supplementary Materials

Summary: The supplementary material consists of three figures.

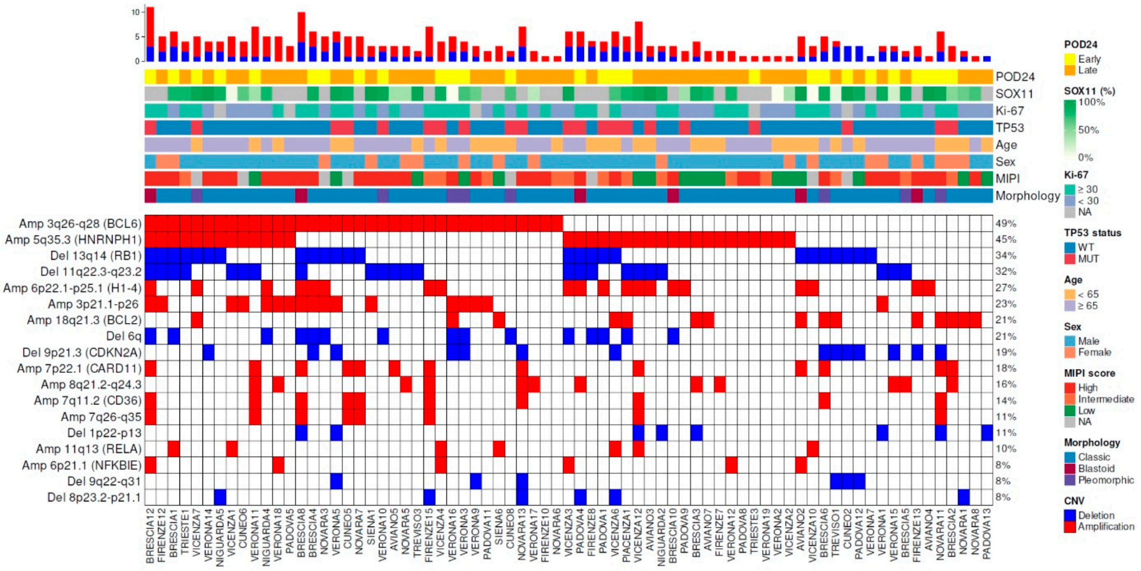

Figure S1. Amplification (red) and deletions (blue) identified in 73 MCL samples (column). On the left the legend annotated the attribution of early (yellow) vs late (orange) POD, SOX11 expression level, *TP53*-WT (blue) and *TP53*-mut (red), age <65 (orange) and ≥65 (purple), sex divided in male (light blue) and female (light orange), MIPI score split into high (red), intermediate (orange), and low (green); and morphology divided in classic (blue), blastoid (dark red) and pleomorphic (purple).

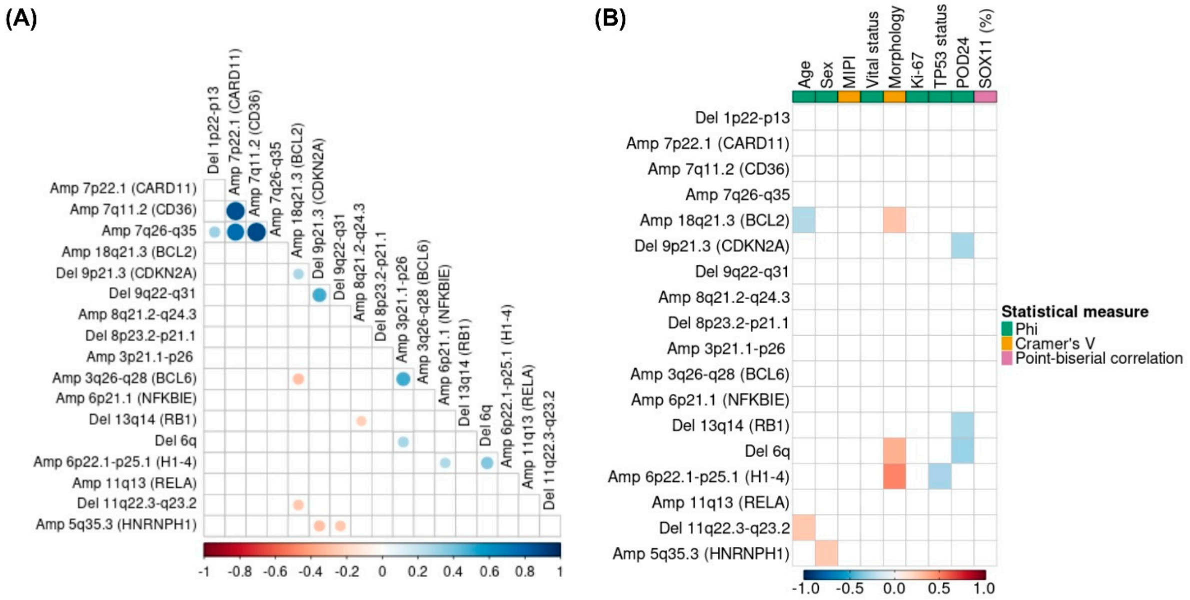

Figure S2. (A) Correlation between CNVs. Pairwise associations between CNVs are shown using the Phi coefficient (color scale, −1 to +1). Only significant correlations (Fisher's exact test, $p < 0.05$) are displayed, while non-significant pairs are left blank. The order on the axes indicates hierarchical clustering of CNVs based on their association profiles. (B) Associations between CNVs and clinical features. Heatmap showing effect sizes for each CNV (rows) versus clinical feature (columns). Colors represent the association measure: Phi for binary–binary tables, Cramer's V for binary–multicategory tables (2×k, k≥3), and point-biserial correlation (r_pb) for binary–continuous comparisons. The color scale is centered at 0 (blue = negative, red = positive; Cramer's V ranges from 0 to 1 and appears on the red side). White cells denote non-significant associations (Fisher's exact test for categorical features; Pearson correlation for continuous features) at $p \geq 0.05$. Column annotations indicate which effect size was used for each feature. The morphology was negatively correlated with Amp 6p22.1-p25.1 (*H1-4*), Amp 18q21.3 (*BCL2*), and Del 6q, while *TP53* mutation was positively correlated with Amp 6p22.1-p25.1 (*H1-4*). In terms of clinical behavior, Del 6q, Del 9p21.3 (*CDKN2A*), and Del 13q14 (*RB1*) showed a positive correlation with early-late POD.

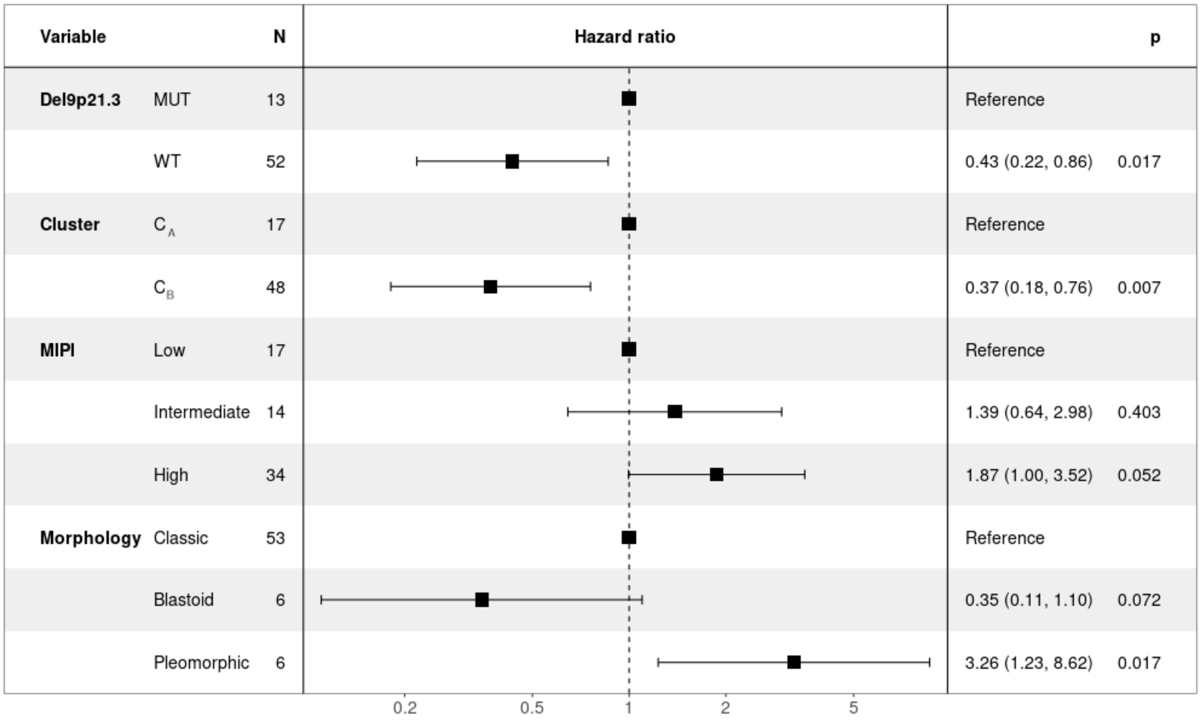

Figure S3. Multivariable Cox regression including CNV-defined cluster assignment. Forest plot reporting HR with 95% confidence intervals and two-sided *p*-values for time to first POD. The initial model included variables significant in univariate analyses, with morphology included a priori; backward stepwise selection was applied. Predictors shown include cluster assignment (C_B compared with C_A), age (≥65 vs <65), Ki-67 (≥30% vs <30%), MIPI (high compared with low/intermediate), morphology (pleomorphic and blastoid compared with classic), Del 9p21.3 (*CDKN2A*), *TP53* mutation, Del 13q14 (*RB1*), and Amp 8q21.2–q24.3.
